# Altitude’s Impact on the Rhizosphere Prokaryotic Communities of the Cretan Endemic Plant *Petromarula pinnata* (L.) A.DC.

**DOI:** 10.3390/microorganisms13010074

**Published:** 2025-01-03

**Authors:** Rafaela Stagiopoulou, Ifigeneia Mellidou, Nikos Krigas, Effimia M. Papatheodorou

**Affiliations:** 1 Department of Ecology, School of Biology, Aristotle University of Thessaloniki, 54124 Thessaloniki, Greece; rafaelastagiopoulou@gmail.com; 2 Institute of Plant Breeding and Genetic Resources, Hellenic Agricultural Organization Demeter (ELGO-Dimitra), 57001 Thessaloniki, Greece; imellidou@elgo.gr (I.M.); or nikoskrigas@gmail.com (N.K.); 3 Department of Viticulture, Floriculture & Plant Protection, Institute of Olive Tree, Subtropical Crops and Viticulture, Hellenic Agricultural Organization Demeter (ELGO-Dimitra), 57001 Thessaloniki, Greece

**Keywords:** soil enzymes, Greece, bacterial communities, chasmophyte, rock dweller, functional diversity

## Abstract

The present study examined the effect of the three different altitudes on the enzymatic activity and the prokaryotic communities of the rhizosphere of *Petromarula pinnata* (L.) A.DC. (Campanulaceae), a vulnerable local endemic species of Crete (Greece). It was observed that the pH and N-acetylglucosaminidase (NAG) activity increased with altitude while the β-1,4-glucosidase (BG) activity fluctuated with increasing altitude. The prokaryotic community in the rhizosphere of *P. pinnata* was dominated at the phylum level by Proteobacteria, Actinobacteriota, Bacteroidota, and Firmicutes, as well as by *Bacillus* members at the genus level. The alpha diversity did not vary with altitude while the *b*-diversity varied significantly, reflecting differences in community composition in relation to altitudinal gradient. The NAG activity was positively associated with most of the predominant phyla, except for Proteobacteria. The BG enzyme activity appeared to be negatively associated with Proteobacteria, Chloroflexi, and Acidobacteriota. Based on online databases, the predicted functions of the community showed a clear distinction in relation to altitude. At lower altitude, functions related to quorum sensing among microbes were overrepresented, while at the higher altitude, the functions were more related to energy production and transfer. The results of this research contribute to the ex situ and in situ protection of the vulnerable populations of *P. pinnata* and provide information for understanding the effect of altitude on processes in the rhizosphere of a threatened local endemic species of Crete studied in its original habitats.

## 1. Introduction

Crete is a Greek island with a long coastline (1046 km) and many mountainous massifs of varied altitudes (highest peak Mt Psiloritis at 2456 m above sea level), a main Mediterranean biodiversity hotspot including over 1800 native plant species, and an important center of endemism with at least 223 local endemic plant species and subspecies [1]. Both its diversified climate and its long evolutionary isolation have allowed for the development of a variety of habitats and high rates of endemism [2,3]. The International Union for the Conservation of Nature (IUCN) has recognized Crete as a Global Centre of Plant Diversity and Endemism [4]. Several Cretan endemic plants are threatened with extinction and many of them have been recognized for their agrifood, medicinal, and ornamental horticultural value [5,6]. Among the Cretan endemic plants, *Origanum dictamnus* L. (Lamiaceae), *Ebenus cretica* L. (Fabaceae), *Petromarula pinnata* (L.) A.DC., and *Campanula pelviformis* Lam. (both Campanulaceae) are autochthonous species that are locally found across a wide range of altitudinal zones [5,7].

Increases in altitude are known to affect the vegetation structure and soil variables such as pH, temperature, precipitation, and soil organic carbon, consequently influencing the biogeochemical cycles of carbon, nitrogen, and phosphorus as well as the soil enzymatic and microbial activities [8,9,10,11]. However, changes in abiotic conditions with altitude tend to vary with confined factors such as local climate, geomorphology, and land use [12,13,14]. In previous studies investigating microbial communities along elevation gradients (e.g., [15]), the *a*-diversity indices decreased with increasing altitude, the microbial interactions became complex at the lowest and highest altitudes, while the functional compositions of the soil bacteria were mainly driven by variations in soil pH and extracellular enzyme activities. On the contrary, other studies (e.g., [16]) have shown that the richness of bacteria, fungi, and viruses may change in a unimodal pattern with increasing elevation. Concerning the role of soil physicochemical variables, previous investigations [16,17] have revealed pH as a key factor for the composition of soil microbial communities across different altitudes. Furthermore, some studies [17] have shown that altitude heightening may increase the oxidation of organic matter and may diversify the rhizosphere microbiome, while others [18] have concluded that not only the taxonomic composition but also the core functional community structure of soil prokaryotic microbes may significantly differ across altitudes. However, such knowledge has never focused on the soil microbiome of local endemic plant species.

The soil microbiome is crucial for maintaining soil fertility since it is related to plant nutrient uptake, plant growth, pathogen resistance, overall plant health, and ecosystem function [19,20,21]. The rhizosphere bacteria and fungi enhance plant resistance to biotic and abiotic stresses such as disease [22,23], high salinity [23,24,25], drought [26], and extreme temperatures [27]. Plant-growth-promoting rhizobacteria (PGPRs) have the potential to produce growth-accelerating phytohormones [28], contribute to nitrogen fixation [26,27], may activate mechanisms that enhance plant resistance against drought, salinity, flooding, and temperature extremes [28,29,30], and enhance plant resistance to stress by preventing metal accumulation [31] or pathogen attack [25,32]. In addition, bacteria belonging to the genera *Azotobacter*, *Bacillus*, *Burkholderia*, *Erwinia*, *Pseudomonas*, *Serratia*, and *Rhizobium* can process inorganic phosphorus, making it available to plant roots [33]. However, plants can both benefit or be negatively affected by microbial populations [20]. The understanding of the interactions between plants and microbial communities provides insights into the ways in which the rhizosphere microbiome enhances plant performance under conditions of biotic and abiotic stresses, like those prevailing at higher altitudes. In this context, knowledge regarding the soil microbiome functions of local endemic plant species is rather absent.

Although several local Cretan endemic species show a wide altitudinal distribution [1], there is no study investigating the effect of altitude on their rhizosphere microbiomes. Therefore, this pivotal study focused on the rhizosphere microbial communities of the threatened local endemic plant of Crete *P. pinnata* [34] along a moderate altitudinal gradient from 45 to 450 m above sea level. Since bacterial and fungal diversity in rhizosphere communities are generally known to be related to plant species identity, vegetation diversity, and below-ground plant traits [35], the investigation of the microbial communities in the rhizosphere of the herein focal species along its range-restricted altitudinal gradient gives a chance to discern specific changes exclusively induced by altitude. The composition, diversity, and predicted functionality of the rhizosphere prokaryotic community was herein studied in relation to soil physicochemical variables and enzyme activity. Such knowledge may provide valuable insights into the ex situ conservation, full acclimatization in anthropogenic settings, and future sustainable utilization of *P. pinnata* as a new multi-purpose crop with significant agroalimentary, nutraceutical/medicinal, and ornamental horticultural value [36]. Understanding the effect of altitude on the performance of a local endemic species of Crete which has naturally adapted to different altitudes may also provide useful information not only for the long-term conservation of this species but also for the conservation of the Cretan plant biodiversity.

## 2. Materials and Methods

### 2.1. Field Sampling and Sample Preparation

*Petromarula pinnata* is a vulnerable (VU) local endemic species of Crete [34] with documented agroalimentary, medicinal/nutraceutical, and ornamental horticultural interest, which is currently under ex situ conservation and in process of domestication in man-made settings [5,6,36,37]. It is a monotypic perennial herbaceous plant, flowering in April and May [7]. It occurs in rock crevices, cliffs, and old walls. It is a medium to tall plant species with a large basal rosette of pinnately lobed leaves and ovate to oblong and coarsely toothed lower leaflets. Across its confined distribution on the island of Crete, this plant species is wild growing at altitudes ranging from 0 to 900 m above sea level [7].

**Figure 1:**
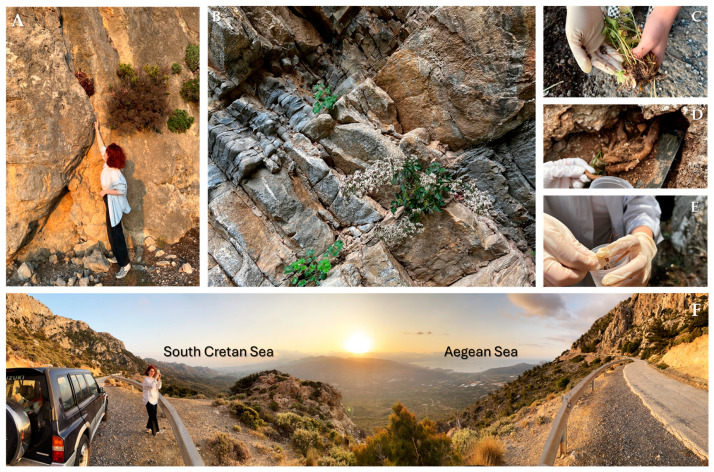
Rocky habitats (**A**,**B**) with mature (flowering) and non-flowering wild-growing individuals of the local endemic *Petromarula pinnata* sampled for above-ground (**C**) and below-ground parts with soil (**D**,**E**) at 450 m above sea level on Mt Thrypti, Crete Island, Greece (**F**) with panoramic view of the Aegean and the South Cretan Seas.

Soil sampling was performed directly from the rhizosphere of *P. pinnata* wild-growing individuals at three different altitudes using a special collection permit issued by the Greek Ministry of Environment and Energy (Permit 38262/2306 of 2/8/2023). The geographical coordinates and the predominant parent material [38] of these sites are presented in Table 1. The lowest altitude in this study was 45 m above sea level in Istron, Lassithi (onwards ‘lower’), the intermediate one was at 260 m in Koudoumi Gorge, and the highest one was at Mt. Trypti at 450 m (onwards ‘higher’) (Figure 1). 

The soil samples were collected in mid-May 2023. At each altitude, five (5) wild-growing individuals of *P. pinnata* were selected and removed together with their root system. At each altitude, the plants were wild-growing individuals with a maximum distance of 10 m between them. The rhizosphere soil that was attached to the root system was removed and collected by shaking the roots by hand while wearing surgical gloves. About 20 g of this material (15 sampled individuals in total) was placed in sterile containers and transferred to the laboratory in a refrigerator at 4 °C. In addition, approximately 200 g of the rhizosphere soil from each plant individual was collected for the determination of soil enzymatic activity and physicochemical variables. All samples were preserved in a portable thermal container while in the field and were transported to the laboratory for processing within 24 h. In the laboratory, the soil samples were homogenized and sieved to study soil physicochemical properties, while an aliquot of each sample was used for DNA isolation.

### 2.2. Determination of Soil Physicochemical Variables

For water content and soil organic carbon (SOC) determination, the samples were oven dried at 105 °C for 24 h (soil moisture measurement), reweighed, and placed in a high-temperature oven for another 16 h at 375 °C (measurement of % organic carbon content).

For soil pH, 20 g of dry soil sample was added to beakers along with 20 mL of deionized water (1:1). A pH meter was used to measure the pH of each sample [39].

### 2.3. Determination of Soil Enzymes

A modified protocol based on a previously published methodology [40] was used to assess N-acetyl-glucosaminidase (NAG), beta-glucosidase (BG), and alkaline phosphatase (AP) activity involved in the N, C, and P cycles, respectively [41]. Fresh soil (1–2 g) was added to 60 mL of 50 mM sodium acetate buffer, pH 5, and was homogenised in a blender for 1 min. Subsequently, 50 mL of homogenised soil slurry was combined with 150 mL of substrate solution and was incubated with constant shaking for 1 h (AP assay), 2 h (BG assays), and 3 h (NAG assay) at 21 °C [42].

The substrate solutions were as follows: 5 mM pNP phosphate for AP, 5 mM pNP-b-glucopyranoside for BG, and 2 mM p-nitrophenyl-N-acetylglucosaminide for NAG. After incubation, 100 mL of the slurry-substrate supernatant was carefully transferred to another microplate for colorimetric determination of product concentrations performed at 450 nm in a spectrophotometer. The enzyme activity of NAG, BG, and AP was expressed in μmolPNP·g^−1^·h^−1^.

### 2.4. DNA Isolation, Sequencing and Data Processing

To isolate the DNA of prokaryotic communities of the rhizosphere of *P. pinnata*, 0.5 g of collected bulk soil from each sample was transferred into phosphate-buffered saline (PBS; 137 nmol L^−1^ NaCl, 1.8 nmol L^−1^ KH_2_PO_4_, 2.7 nmol L^−1^ KCl and 1.42 nmol L^−1^ Na_2_HPO_4_, pH = 7.4) and was sonicated for 10 min. Then, the solutions were subsequently centrifuged at 10,000 rpm for 20 min, and the sedimentation material was placed in −80 °C until further use [43]. DNA was extracted from a total of 15 samples using the NucleoSpin^®^ DNA isolation kit according to the manufacturer’s instructions (Macherey-Nagel, Dueren, Germany). DNA concentration and quality was estimated using a NanoDropTM spectrophotometer (Thermo ScientificTM, Waltham, MA, USA) and was confirmed via gel electrophoresis. After the estimation of DNA concentration and extraction, we kept three samples per altitude (those with the higher DNA quality) for further analyses.

The isolated DNA was subjected to PCR using the specific primers targeting the V3–V4 hypervariable region of the 16S rRNA gene (341F: 5′-CCTAYGGGRBGCASCAG-3′, 806R: 5′-GGACTACCVGGGTATCTAAT-3′′). The amplified 16S rRNA amplicons from each sample were paired-end sequenced (2 × 250) on the Illumina NovaSeq 6000 platform in accordance with the standard protocol. The PCR amplification and sequencing steps were performed at Novogene (UK) Company Ltd. (25 Cambridge Science Park, Milton Road, Cambridge, UK). The raw sequence data were submitted to the NCBI SRA database (NCBI BioProject PRJNA1190203).

For data processing, the raw paired-end reads were assigned to samples based on their unique barcodes and were merged using FLASH (V1.2.7) [44]. The tags were then compared with the reference database of SILVA 138 (https://www.arb-silva.de/, accessed on 1 September 2024) to detect chimera sequences, while the effective tags were obtained by removing the chimera sequences with the search package V2.16.0 [45]. DADA2 was used to reduce noise and extract amplicon sequence variant (ASV) sequences [46]. All ASV sequences were annotated using the QIIME2 software (https://forum.qiime2.org/, accessed on 13 September 2024). The top ten taxa (taxonomic units) of each sample were selected to plot the distribution histogram of relative abundance at the phylum and genus levels. The absolute abundance of ASVs was normalized using a standard sequence number corresponding to the sample with the fewest sequences.

### 2.5. Statistical Analyses

A one-way ANOVA followed by a Tukey HSD test was applied in SPSS Statistics v.27.0 (SPSS, Inc., IBM Corp., Armonk, NY, USA) to determine the effect of altitude on enzyme activity and soil physicochemical characteristics. Analysis of the alpha diversity and beta diversity of the rhizosphere microbial communities were performed based on the output normalized data in QIIME2. The nonparametric Kruskal–Wallis test was employed to determine if there were statistically significant differences in diversity indices between the three altitudes. Principal coordinate analysis (PCoA) was performed to generate principal coordinates based on the Jaccard index using the permutational multivariate analysis of variance (PERMANOVA) statistical method. To characterize the microbial differences between the various groups, and to identify potential biomarkers for each altitude, linear discriminant analysis (LDA) effect size (LEfSe) analysis was employed using MicrobiomeAnalyst 2.0 web-based tool [47], with the LDA threshold set at 3. Finally, PICRUSt2 was employed to predict the metagenomic functions based on marker genes using the COG database [48]. Further, we analyzed the correlations between the abundance of the top-represented phyla, the soil physicochemical variables, and the enzyme activity.

## 3. Results

### 3.1. Soil Physicochemical Variables and Enzymatic Activities

**Figure 2:**
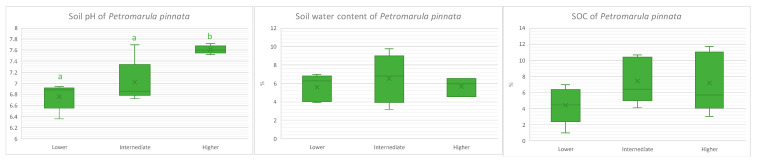
Soil characteristics (pH, water content, and organic carbon) of *Petromarula pinnata* at three different altitudes in eastern Crete. The letters a and b indicate the statistically significant differences between altitudes.

**Figure 3:**
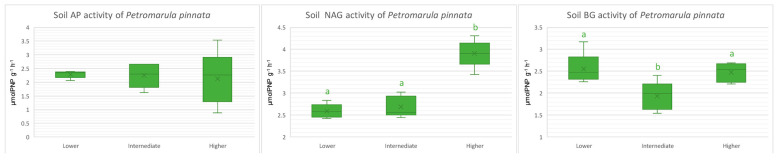
Enzyme activity (AP: alkaline phosphatase, NAG: N-acetylglucosaminidase, BG: β-1,4-glucosidase) in the rhizosphere soil of *Petromarula pinnata* at three different altitudes in eastern Crete. The letters a and b indicate statistically significant differences with respect to altitude as obtained by Tukey test.

Altitude affected significantly only pH, while water content and SOC were not affected by elevation. In fact, pH at the higher altitude was significantly higher than at the other two altitudes (Figure 2). Also, altitude had a significant effect on the activity of NAG and BG and not on AP. The highest NAG activity was recorded at the higher altitude and was significantly different from that at the other two altitudes, while the lowest BG was recorded at the intermediate altitude (Figure 3) and was significantly different than the other two altitudes. The results of the one-way ANOVA and Tukey HSD test for result significant are available in the Supplementary Materials Tables S1 and S2.

### 3.2. Community Composition and Function

#### 3.2.1. Relative Abundance

**Figure 4:**
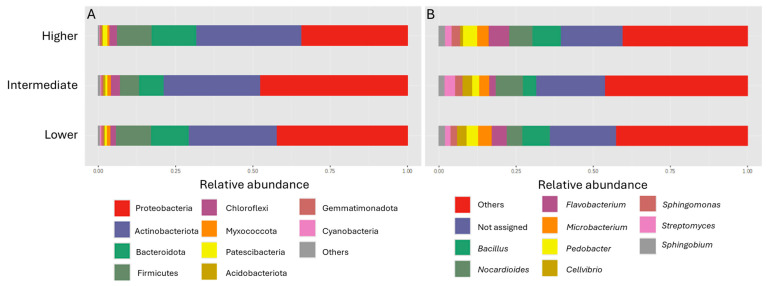
Relative abundance of the top ten most abundant taxa of *Petromarula pinnata* rhizosphere samples at different altitudes in eastern Crete at the levels of phylum (**A**) and genus (**B**).

At the phylum level and regardless of altitude, it was observed that the three most predominant phyla were Proteobacteria, Actinobacteriota, and Bacteroidota, followed by Firmicutes and Chloroflexi (Figure 4A). Taxa abundancies at the phylum and genus levels are presented in Supplementary Table S3. The relative abundance of Actinobacteriota increased with the heightening altitude, whereas Proteobacteria and Bacteroidota were more and less abundant, respectively, at the intermediate altitude. The relative abundance of Proteobacteria was 27.43% at the lower altitude, 36.26% at the intermediate, and 30.58% at the higher one. Accordingly, the relative abundances of other phyla at the lower, intermediate, and higher altitudes, respectively, were 11.98%, 13.30%, and 19.95% for Actinobacteriota, 4.78%, 3.90%, and 8.96% for Bacteroidota, and 4.50%, 3.38%, and 6.44% for Firmicutes. Other phyla with lower abundances that were present at all altitudes included Chloroflexi, Acidobacteriota, Patescibacteria, and Gemmatimonadota. The results of the ANOVA analysis of the relative abundances at the phylum level demonstrated significant differences between the three altitudes with regard to Proteobacteria, Bacteroidota, and Patescibacteria (Supplementary Table S2). In particular, Proteobacteria (*p* < 0.001) were more abundant at the intermediate altitude, whereas Bacteroidota (*p* < 0.05) and Patescibacteria (*p* < 0.05) were more abundant at the higher altitude.

At the genus level, the three most predominant genera were *Bacillus*, *Nocardioides*, and *Flavobacterium*, followed by *Microbacterium* and *Pedobacter* (Figure 4B). The relative abundance of *Bacillus* was 0.09% at the lower altitude, 0.04% at the intermediate altitude, and 0.09% at the higher altitude. Accordingly, the relative abundances of the other genera at the lower, intermediate, and higher altitudes, respectively, were 0.05%, 0.08%, and 0.07% for *Nocardioides*, 0.05%, 0.02%, and 0.07% for *Flavobacterium*, and 0.04%, 0.03%, and 0.03% for *Microbacterium*. Other genera with lower relative abundances included *Sphingomonas*, *Sphingobium*, and *Streptomyces*. An interesting note is that *Flavobacterium* was positively correlated with the higher altitude’s samples.

#### 3.2.2. Alpha and Beta Diversity

**Figure 5:**
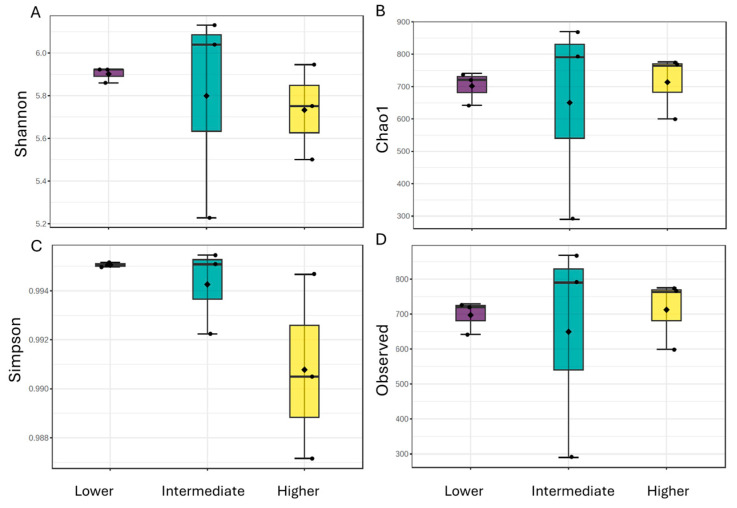
Alpha diversity indices ((**A**) Shannon index, (**B**) Chao1, (**C**) Simpson and (**D**) Observed taxa) in the rhizosphere microbial communities of *Petromarula pinnata* in relation to altitude.

Alpha diversity in the rhizosphere microbial communities of *P. pinnata* as measured by Shannon and Simpson indices displayed a decreasing trend with increasing altitude (Figure 5). In particular, the Shannon index decreased from 5.85 at the lower altitude to 5.67 at the higher altitude, while the Simpson index decreased from 0.995 to 0.990. However, neither index showed statistically significant differences in relation to altitude. Similarly, Chao1 and the number of observed taxa did not exhibit significant variations across the different altitudes. Therefore, while there was a tendency toward decreasing diversity and richness at higher altitudes, this trend was not statistically significant. Notwithstanding the lack of statistically significant differences, the variability was most prominent at the intermediate altitude across all indices, indicating a higher environmental heterogeneity at this altitude, leading to a wide range of richness and diversity values. By contrast, the lower and, to a lesser extent, higher altitudes showed relatively narrower ranges, suggesting more uniform microbial communities.

**Figure 6:**
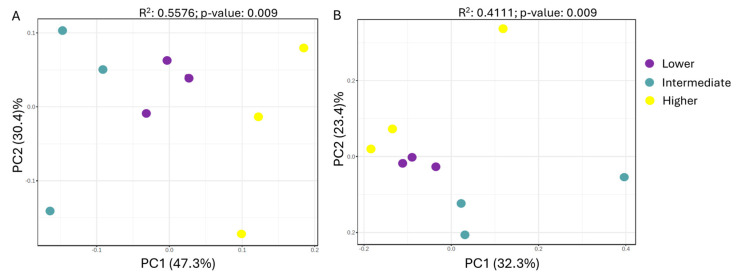
Principal coordinate analysis (PCoA) of the rhizosphere microbial communities of *Petromarula pinnata* recorded at three different altitudes in eastern Crete, based on the Jaccard dissimilarity index, at the phylum (**A**) and the genus (**B**) levels.

Regarding beta diversity, the principal coordinate analysis (PCoA) revealed moderate differences regarding altitude, at both the phylum and genus levels (Figure 6). The two principal components were able to discern the three altitudinal classes, with an overall contribution of 77.7% and 55.7% at the phylum and genus levels, respectively. The lower altitude showed a lower variation in community composition between samples (Figure 5). PERMANOVA analysis performed at the levels of phylum (F-value: 3.7813, R^2^: 0.5576, *p*-value: 0.009) and genus (F-value: 2.0943, R^2^: 0.4111, *p*-value: 0.009) revealed significant differences between the bacterial community composition of the three altitudinal classes, probably reflecting the distinct ecological and/or environmental conditions.

#### 3.2.3. LEfSE Analysis and Functional Prediction

**Figure 7:**
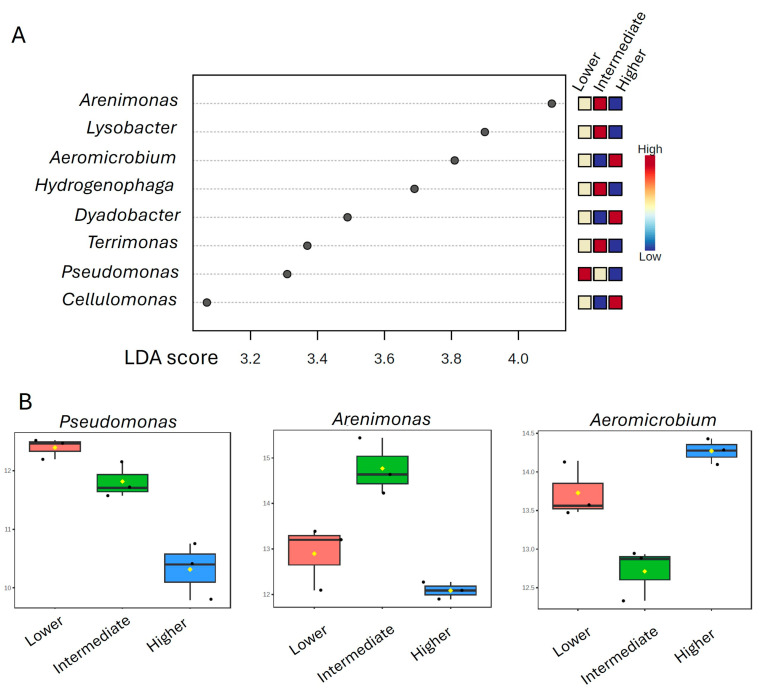
(**A**) LEfSE analysis of the rhizosphere microbial communities of *Petromarula pinnata* at the genus level, at three different altitudes in eastern Crete. The LDA threshold was set at 3 and the *p*-value cut-off at 0.05. The dots represent the genera with statistical differences among the three altitudinal classes. Different colors represent the relative abundances of the specific genera in each altitude (red for high and blue for low abundances). (**B**) Log-transformed counts of putative microbial biomarkers for the lower, intermediate, and higher altitudes.

The LEfSe analysis detected several genera in the microbial rhizosphere communities of *P. pinnata* as putative biomarkers for the three altitudinal classes (Figure 7). In particular, candidate biomarkers for the lower altitude included *Pseudomonas*; for the intermediate altitude, the genera *Arenominas*, *Lysobacter*, *Hydrogenophaga*, and *Terrimonas*; and for the higher altitude, the genera *Aeromicrobium*, *Dyadobacter*, and *Cellulomonas*. These potential biomarkers were associated with each altitude where *P. pinnata* was wild growing in eastern Crete.

**Figure 8:**
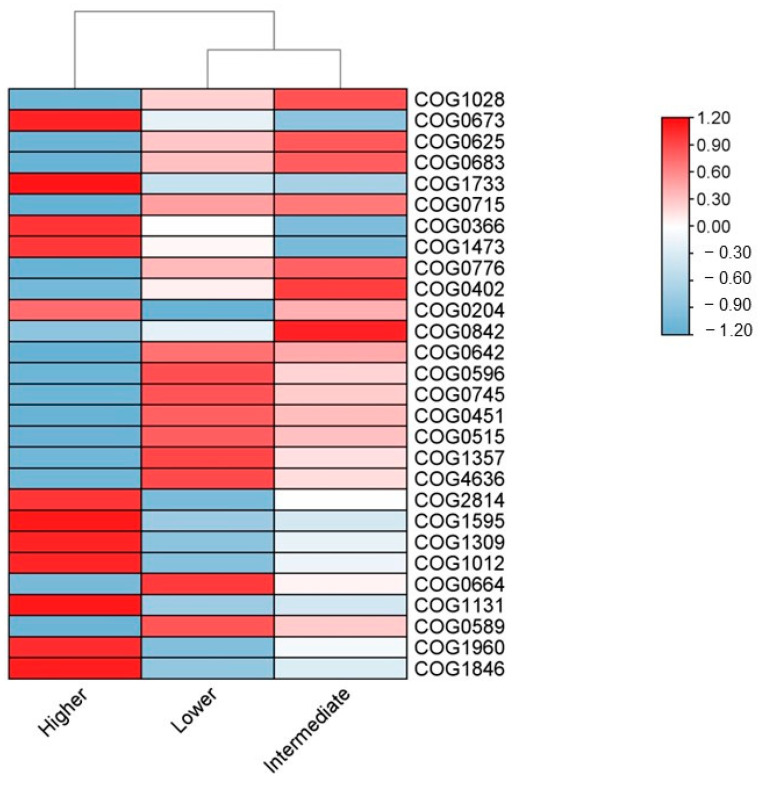
Heatmap showing predicted functions according to the COG library for prokaryotic communities in the rhizosphere of *Petromarula pinnata* at three different altitudes in eastern Crete. Only those showing significant differences in pairwise comparisons (*p* < 0.05) are presented. More details are presented in Supplementary Materials Table S3.

The functional characteristics of *P. pinnata* rhizosphere microbial communities were studied at the protein level using the COG library (COG 2024) (Figure 8). The results indicate that the lower and the intermediate altitudes were grouped closer compared to the higher altitude based on their predicted functions. The samples of the lower altitudinal class had a decreased presence of a 1-acyl-sn-glycerol-3-phosphate acyltransferase (COG0204) involved in the lipid transport and metabolism pathway, a protein-related ABC-type multidrug transport system, a permease component (COG0842) involved in the defense pathway, and a cytosine/adenosine deaminase or related metal-dependent hydrolase (COG0402) from the nucleotide transport and metabolism pathway, compared to the intermediate altitude (Figure 8; Supplementary Table S3). When comparing the intermediate and higher altitudes, a NAD(P)-dependent dehydrogenase (COG1028; lipid and transport pathway), proteins related to an ABC-type branched-chain amino acid transport system (COG0683; amino acid transport) or to an ABC-type nitrate/sulfonate/bicarbonate transport system (C0G0715; transcription), a bacterial nucleoid DNA-binding protein (COG0776; replication, recombination and repair), and a cytosine/adenosine deaminase (COG0402; nucleotide transport and metabolism) were more abundant at the intermediate altitude. By contrast, a glutathione S-transferase (COG0625; post-translational modification), a DNA-binding transcriptional regulator of the HxlR family (C0G1733; transcription), and a glycosidase (COG0366; carbohydrate transport and metabolism) were enriched at the higher altitude. The highest number of proteins differing between the three altitudinal classes were observed in pairwise comparisons between the lower and higher altitudes. In particular, a nucleoside-diphosphate-sugar epimerase (COG0451) and several proteins related to signal transduction mechanisms (COG0642, COG0515, COG0664, COG0589) were found upregulated at the lower altitude. Accordingly, an acyl-CoA reductase (COG1012; energy production and conversion), an arabinose efflux permease of the MFS family (COG2814; carbohydrate transport), an acyl-CoA dehydrogenase related to the alkylation response protein AidB (COG1960L lipid transport and metabolism), an ABC-type multidrug transport system (COG1131; defense mechanisms), as well as several proteins related to transcription (COG1595; COG1309; COG1846) were found to be enriched at the higher altitude. Collectively, our results highlight the greatest functional divergence between the lower and higher altitudes, underscoring significant altitudinal influences on microbial community functions.

#### 3.2.4. Correlation Between Community Composition, Soil Physicochemical Variables and Enzyme Activity

**Figure 9:**
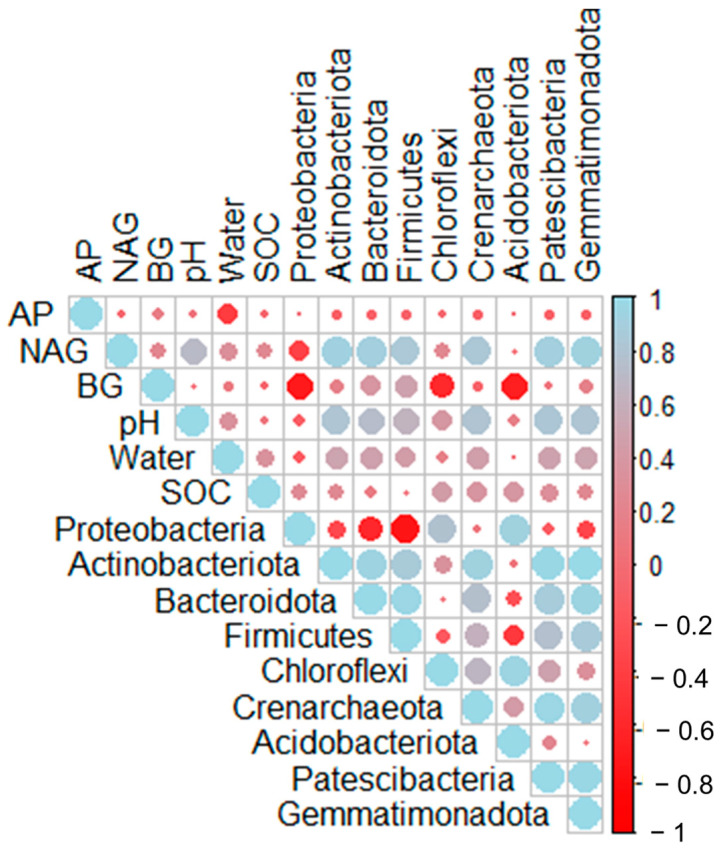
Correlation analysis between the relative abundance of the dominant phyla, soil characteristics, and enzyme activities recorded in the microbial communities of the rhizosphere of *Petromarula pinnata*.

Remarkably, no relation between enzymes and SOC was recorded. Also, there was no correlation between the SOC content and the composition of the community at the phylum level. On the contrary, pH showed a strong positive correlation with most phyla except Proteobacteria and Acidobacteriota. Concerning enzymes, NAG was positively correlated to most phyla except Proteobacteria and Acidobacteriota, whereas BG appeared to be negatively related to Proteobacteria, Chloroflexi, and Actinobacteriota (Figure 9).

## 4. Discussion

*Petromarula pinnata* has strong agroalimentary, medicinal/nutraceutical, and ornamental horticultural interest [5,6,36,49,50]. Despite the intense research interest concerning the ex situ conservation conditions of *P. pinnata* as a vulnerable species and its germination capacity [49], almost nothing is known about its soil physicochemical environment, the activity of soil enzymes, and its rhizospheric prokaryotic communities, or about the effect of altitude on these variables. This investigation attempted to facilitate the ongoing conservation efforts of *P. pinnata* and its sustainable exploitation actions by filling these gaps.

Increasing altitude has been observed to affect abiotic conditions such as pH, temperature, precipitation, soil and vegetation organic carbon, biogeochemical cycles of carbon, nitrogen, and phosphorus [8,9,10], as well as soil enzyme and microbial activity [11]. However, changes in abiotic conditions with altitude tend to vary with local factors such as local climate, geomorphology, and land use [12,13,14,51]; there is no specific trend underlying the influence of altitude on these factors at a general level or specifically for the island of Crete, beyond the natural phenomenon of temperature decrease at higher altitudes.

The soil water content in the rhizosphere soil of *P. pinnata* was rather similar along the altitudinal gradient examined, although precipitation at the higher altitude is on average 30% higher than at the lower one and the air temperature is about 3 °C lower [52]. The stability of water content and organic carbon across altitudes in this study could be related to the fact that the samples were taken exclusively from the rhizosphere of *P. pinnata* and not from the surrounding vegetation that changes along an altitudinal gradient. Furthermore, the effect of surrounding vegetation was negligible since this species grows almost exclusively as a chasmophyte (rock dweller), creating its own microenvironment. In this context, we hypothesized that the altitudinal gradient was too narrow (45 to 450 m) to result in large differences in terms of physicochemical soil properties such as water content and SOC. In the same fashion, another study [53] has shown that total organic carbon and total nitrogen in the soil of broadleaf red pine forests do not change in an elevation gradient from 700 to 1045 m. On the contrary, pH in the rhizosphere soil of *P. pinnata* increased significantly with altitude from 6.5 to 7.5. In general, pH does not follow a specific response pattern in relation to altitude but may depend on the parent rock and local vegetation [54]. Since in this study the vegetation was almost identical (phrygana and low shrub communities), the pH changes were probably related to parent material or to rhizosphere effects; the roots of *P. pinnata* at the higher altitude probably were able to release more H+ compared to the amounts of H+ released at the low and intermediate altitudes (it is well established that plants secrete OH− or H+ in their root environment to keep a balance between the amounts of cations and anions in their issues). Interestingly, pH proved to be the only driver of the microbial community composition, exhibiting significant positive correlation with most bacterial phyla except Chloroflexi and Acidobacteriota, although its range of variation was rather narrow. Frequently, pH has been acknowledged as a critical factor regulating bacterial communities across various spatial scales [16,23,55]. Furthermore, by employing a partial Mantel test, we identified soil pH as the driver of the predicted functional profile of the microbial communities as well.

The activities of BG and NAG were affected significantly by altitude in the case of the rhizosphere of *P. pinnata*. Soil from the intermediate altitude exhibited the lowest BG activity, while there were no differences between the lower and the higher altitude. BG enzyme activity is generally associated with the degradation of the labile forms of organic material (particularly cellulose) [56], while NAG activity, which in this study increased at the higher altitude, is known to be related to the degradation of chitin present in the cell walls of fungi and arthropods and is strongly influenced by fungi, soil pH, and nutrient availability [56]. In the case investigated herein, the increase of pH with altitude probably resulted in an increase in NAG activity. Although it was expected that the relationship of enzymes with elevation would have been like the relationship of organic carbon with elevation (given that enzymes are tied to soil organic material), this was not the case.

The analysis of the composition of the rhizosphere prokaryotic communities of *P. pinnata* in relation to altitude revealed significant differences. The significant differences in *b*-diversity suggested that although the within-community diversity (*a*-diversity) was similar, the community composition differed between the three altitudinal classes, suggesting either that the altitude, by affecting the quality of root exudates [57], had a strong effect over unique taxa composition or that specific taxa may dominate at a certain altitude but be absent at another. Since the differences concerning the most abundant phyla and genera were minor, the differences in *b*-diversity seem mostly to be due to rare taxa. No effect of elevation on bacterial diversity was similarly reported in other investigations [58] examining the soil bacterial communities on an altitudinal gradient from 700 to 1000 m under identical vegetation. The most abundant bacterial phyla along the altitudinal gradient were Proteobacteria, Acidobacteriota, Bacteriota, and Firmicutes, and their relative abundance tended to increase at the higher altitude. However, only Bacteroidota, Proteobacteria, and Patescibacteria exhibited significant differences in relation to altitude. Proteobacteria were abundant at the intermediate altitude; Bacteroidota and Patescibacteria at the higher altitude. Microbes are recognized as r- or K-strategists depending on their life history traits. Under conditions of resource limitation, K-strategist microbes exhibiting relatively slow growth rates predominate, while in more unstable environments with high resource availability, the predominance shifts to r-strategist microbes [59]. Proteobacteria and Bacteroidota, considered copiotrophic (r-strategists), generally exhibited higher relative abundances in the surface soil, thriving in soils with abundant labile SOC and nutrients [59,60]. The latter coupled with the fact that the precipitation at the higher altitude was on average 750 mm per year [52] gave grounds to assume that at this altitude the productivity of *P. pinnata* was relatively high and supported fast nutrient recycling. Such nutrients were probably used by local r-strategist microbes such as Bacteroidota.

Contrary to community composition, the analysis of the predicted functions according to the COG library revealed distinct differences in the predicted functionality of the rhizosphere microbial communities of *P. pinnata* along the altitudinal gradient, grouping the lower and intermediate altitudes closer to each other compared to the higher altitude. The COG codes examined herein could be categorized in 17 functional categories (https://www.ncbi.nlm.nih.gov/research/cog/# (accessed on 30 December 2024)). According to this categorization, the COG codes at the lower altitude corresponded to functions that were related mainly to cellular health (lipid transport and metabolism, cell wall biogenesis) and the communication between bacteria (signal transduction mechanisms), often called quorum sensing. Bacterial quorum sensing in salt-affected environments, like those at the low altitude examined herein, seem to promote biofilm formation, osmoregulation, nutrient acquisition, and community defense. This communication strategy probably enables bacterial populations to act as cohesive communities, enhancing *P. pinnata*’s survival and resilience in harsh ecosystems. Indeed, a quorum sensing system has been evidenced as an essential function in the response of plant-growth-promoting rhizobacteria (PGPR) to environmental stress and PGPR induction of plant tolerance to saline–alkaline stress [61,62].

In the higher altitudinal class, prokaryotic predicted functions related to the transport and metabolism of amino acids, nucleotides, and lipids, and to the transcription process to gain energy to produce proteins such as enzymes that are necessary for the decomposition of organic materials. Bacterial metabolism is a complex, highly regulated process that enables bacteria to adapt rapidly to changing environmental conditions by directing limited resources to functions needed the most. For instance, some studies have observed a down-regulation of the bacterial protein biosynthesis machinery in warmed soils [63], coinciding with lower microbial biomass, RNA, and soil substrate content. Sollinger and colleagues therefore concluded that down-regulation of the protein biosynthesis machinery liberates energy and matter, allowing soil bacteria to maintain high metabolic activity and cell division rates, even after decades of warming. In this way, the predicted microbial functions at the higher altitude in this study were related mostly to the biosynthesis of proteins rather than cell division or quorum sensing.

Differences in functional profiles of microbial communities along an altitudinal gradient have been repeatedly reported in the literature [16,18,58], although not for local endemic plant species as referred to herein. In these studies, the changes in functional profiles coincided with changes in taxonomic community structure. Although local diversity within the three altitudinal classes examined in this investigation was similar, the specific taxa present along with their predicted functions differed significantly, highlighting significant changes in community composition, probably reflecting environmental or ecological gradients influencing taxa distribution without drastically altering local biodiversity.

## 5. Conclusions

This paper is the first study that has examined the effect of altitude on soil characteristics, soil enzyme activity, and rhizosphere prokaryotic communities of *P. pinnata*, a vulnerable local endemic species of Crete, Greece. An increase in altitude from 45 to 450 m induced increases in pH values and NAG activity, while soil water content, SOC, and AP activities were unaffected. BG responded to the altitudinal gradient, but not monotonically. However, the relative abundance of Proteobacteria and Bacteroidota, which are r-strategists, increased at the intermediate and higher altitudes, providing evidence for faster nutrient recycling compared to the lower altitude. The observed differences in *b*-diversity suggest that environmental factors affecting the quality of root exudates in each altitudinal habitat were probably the key drivers in shaping taxa presence and abundance along the three classes, even though the overall level of diversity within each site (*a*-diversity) remained relatively similar. The differences in community composition seem to be related to differences in function: at the lower altitude, the community functions were linked to quorum sensing among microbes, while at the higher altitude they were related to energy production and transfer.

In conclusion, this research provides an insight into the effect of altitude on a local Cretan endemic Campanulaceae species with conservation concern and sustainable utilization potential, thus encouraging further study of the effect of altitude on other endemic and native species of Crete to obtain a deeper insight into the protection of the invaluable local biodiversity.

## Figures and Tables

**Table 1:** Geographical coordinates, altitudes, and predominant bedrock of the sampling sites.

Area	Latitude	Longitude	Altitudinal Class	Altitude (m)	Prevailing Parent Material
Istron	35°07′29.2″ N	25°44′55.5″ E	Lower	45	Tertiary deposits
Koudoumi	35°09′060″ N	25°56′01.7″ E	Intermediate	260	Hard limestones and shales
Thrypti	35°04′020″ N	25°49′36.0″ E	Higher	450	Μixed flysch

## Data Availability

The raw sequence data were submitted to the NCBI SRA database (NCBI BioProject PRJNA1190203).

## Supplementary Materials

The following are available online at https://www.mdpi.com/article/10.3390/microorganisms13010074/s1, Supplementary material Table S1: Results of parameters’ comparisons by One Way ANOVA in the soil of *Petromarula pinnata*; Table S2: Results of Tukey HSD parameters’ comparisons in the soil of *Petromarula pinnata*; Table S3: Raw counts of taxonomic units at the phylum and genus levels per altitudinal class (Lower: 45 m; Intermediate: 260 m; Higher: 450 m above sea level); Table S4: Analysis of variance (ANOVA) for the relative abundances of the top 10 phyla and genera (*** *p* < 0.001; * *p* < 0.05); Table S5: T-test of significance on the predicted functions according to the COG library for prokaryotic communities in the rhizosphere of *Petromarula pinnata* at three different altitudes in eastern Crete (** *p* < 0.01; * *p* < 0.05).
