# Supplementary material for: Altitude’s Impact on the Rhizosphere Prokaryotic Communities of the Cretan Endemic Plant Petromarula pinnata (L.) A.DC
Source: Microorganisms. 2025 Jan 3;13(1):74. doi: 10.3390/microorganisms13010074 (PMC11767782; doi:10.3390/microorganisms13010074)
Supplement: Supplementary file 1 [file microorganisms-13-00074-s001.zip › microorganisms-3393407-supplementary.pdf]

Table S1. Results of parameters' comparisons by One Way ANOVA in the soil of *Petromarula pinnata*

**Parameter**		**Sum of squares**	**df**	**Mean square**	**F**	***p***
NAG	Between groups	5.322	2	2.661	43.606	0
	Within the groups	0.732	12	0.061		
	Total	6.054	14			
BG	Between groups	1.131	2	0.565	6.016	0.015
	Within the groups	1.128	12	0.094		
	Total	2.259	14			
pH	Between groups	1.89	2	0.945	13.272	0.001
	Within the groups	0.854	12	0.071		
	Total	2.744	14			

Table S2. Results of Tukey HSD parameters' comparisons in the soil of *Petromarula pinnata*

	**Dependent variable**		**Standard deviation**	***p***	**Confidence intervals**	
NAG	Lower	Intermediate	0.156	0.82	-0.511	0.322
		Higher	0.156	0*	-1.725	-0.891
	Intermediate	Lower	0.156	0.82	-0.322	0.511
		Higher	0.156	0*	-1.63	-0.797
	Higher	Lower	0.156	0*	0.891	1.725
		Intermediate	0.156	0*	0.797	1.63
BG	Lower	Intermediate	0.194	0.02*	0.1	1.135
		Higher	0.194	0.916	-0.439	0.595
	Intermediate	Lower	0.194	0.02*	-1.135	-0.1
		Higher	0.194	0.041*	-1.057	-0.022
	Higher	Lower	0.194	0.916	-0.595	0.439
		Intermediate	0.194	0.041*	0.022	1.057
pH	Lower	Intermediate	0.169	0.313	-0.708	0.192
		Higher	0.169	0.001	-1.298	-0.398
	Intermediate	Lower	0.169	0.313*	-0.192	0.708
		Higher	0.169	0.011*	-1.04	-0.14
	Higher	Lower	0.169	0.001*	0.398	1.298
		Intermediate	0.169	0.011*	0.14	1.04

* The mean difference is statistically significant at 0.05.

Table S3. Raw counts of taxonomic units at the phylum and genus levels per altitudinal class (Lower: 45 m; Intermediate: 260 m; Higher: 450 m above sea level).

**Altitude**	45 m	45 m	45 m	260 m	260 m	260 m	450 m	450 m	450 m
**Phyla**									
Acidobacteriota	106	117	104	37	294	138	28	241	117
Actinobacteriota	3840	3149	4398	2200	4889	5142	6827	5434	6629
Armatimonadota	7	12	17	6	18	21	16	44	29
Bacteroidota	1338	1754	1519	597	1797	1076	2389	3405	2390
Chloroflexi	162	247	357	73	915	712	220	705	660
Cyanobacteria	80	99	78	63	39	45	85	64	73
Firmicutes	1110	1201	2043	60	1667	1354	1196	2466	2449
Gemmatimonadota	113	77	98	20	171	82	26	220	177
Halobacterota	5	0	0	0	9	25	0	4	0
Latescibacterota	6	1	3	0	2	0	0	2	3
Methylomirabilota	5	3	6	0	6	7	0	15	10
Myxococcota	163	197	139	88	229	165	13	216	88
NB1_j	4	5	7	0	7	7	0	2	0
Nitrospirota	10	9	9	4	29	20	9	3	16
Not_Assigned	12	10	13	0	11	16	9	23	28
Others	3	0	2	0	11	5	2	6	7
Patescibacteria	56	58	57	41	167	72	282	170	329
Planctomycetota	2	1	0	6	1	1	0	0	0
Proteobacteria	5131	5380	6253	2882	10510	7837	6332	6126	6976
Verrucomicrobiota	10	19	15	32	14	24	6	36	7
WS2	0	1	4	0	2	2	0	4	0
**Genera**									
Achromobacter	4	0	5	0	6	6	6	0	9
Acidibacter	40	72	42	0	153	73	0	37	26
Acidothermus	0	0	0	0	0	0	0	10	7
Acidovorax	62	59	61	0	105	56	187	0	9
Acinetobacter	12	29	41	0	28	3	27	14	19
Actinocorallia	2	2	9	0	0	0	6	17	7
Actinomadura	0	7	15	0	17	0	0	9	9
Actinomycetospora	8	0	7	0	0	0	9	14	0
Actinophytocola	0	0	5	0	7	0	0	9	3
Actinoplanes	4	5	5	8	20	15	0	0	0
Actinopolymorpha	4	4	0	0	9	4	0	6	6
Adhaeribacter	0	0	0	4	13	4	11	4	11
Aeromicrobium	221	141	181	46	105	124	349	350	441
Aeromonas	4	5	0	0	12	23	0	0	0
Agrococcus	10	0	0	12	0	14	24	7	15
Agromyces	158	137	151	76	263	248	258	169	213

Ahniella	0	8	4	5	16	9	0	0	0
Algoriphagus	20	17	17	52	18	12	24	31	52
Allorhizobium_Neorhizobium_Pararhizobium_Rhizobium	147	248	271	32	205	174	1164	220	337
Altererythrobacter	50	40	37	84	124	104	71	33	91
Amaricoccus	56	88	98	4	89	131	85	121	159
Ammoniphilus	27	19	29	0	41	20	22	39	33
Arachidicoccus	0	0	0	0	0	0	0	10	9
Arcticibacter	0	0	1	0	0	0	22	1	0
Arenibacter	0	0	0	0	8	0	0	4	6
Arenimonas	131	57	144	262	518	318	86	76	90
Aridibacter	0	6	4	0	10	7	0	0	3
Arthrobacter	32	45	49	0	19	38	56	52	62
Asticcacaulis	50	49	66	18	71	32	15	177	136
Bacillus	905	985	1608	53	1230	999	894	2091	2060
Bauldia	19	11	0	0	22	10	0	0	11
Bhargavaea	3	3	2	0	0	4	0	0	0
BIyi10	3	5	0	0	6	7	0	4	0
Blastococcus	11	42	67	31	15	72	92	78	72
Bosea	29	46	31	6	136	76	69	53	72
Brachybacterium	0	4	11	14	0	2	5	8	11
Bradyrhizobium	22	31	49	22	131	79	29	43	45
Brevundimonas	14	37	40	12	53	50	34	25	106
Bryobacter	27	0	14	10	32	19	0	26	10
Caenimonas	0	0	0	20	11	10	0	0	0
CandidatuAlysiosphaera	10	8	21	0	26	14	22	5	26
CandidatuChloroploca	5	0	7	0	0	5	2	0	7
CandidatuSolibacter	2	3	0	0	0	6	0	0	3
CandidatuUdaeobacter	0	2	0	0	0	0	0	11	4
Catellatospora	0	0	0	0	12	16	14	0	20
Caulobacter	95	133	133	89	103	104	149	135	149
Cellulomonas	26	15	30	0	14	0	50	43	51
Cellulosimicrobium	34	27	39	0	23	33	34	34	27
Cellvibrio	462	428	412	262	765	363	67	239	343
Chiayiivirga	6	5	5	47	19	8	7	0	0
Chitinophaga	3	17	23	2	20	27	12	49	38
Chryseobacterium	46	63	71	0	30	19	2	70	108
Chryseolinea	13	12	27	2	25	11	9	18	18
Citricoccus	13	18	27	0	29	26	13	28	24
CL500_29marinegroup	9	10	7	5	9	8	0	7	4
Clostridiumsensustrict1	7	13	12	0	45	30	10	22	14
Cohnella	0	0	0	0	12	6	9	0	0
Conexibacter	17	10	7	0	0	2	0	10	16
Craurococcus_Caldovatus	0	0	0	0	4	0	0	2	5
Crocinitomix	0	0	0	8	0	2	5	0	0
Cytophaga	0	0	0	0	0	0	0	19	27
Dactylosporangium	4	3	0	0	4	0	0	5	7
Devosia	190	155	198	163	399	319	391	305	410
Dokdonella	95	84	134	25	115	66	30	128	69

Domibacillus	7	0	9	0	0	13	12	0	13
Dongia	70	71	69	72	172	134	22	115	91
Dyadobacter	33	63	45	11	29	27	94	240	113
Edaphobaculum	0	9	7	0	30	18	1	13	3
Ellin6067	4	0	0	0	13	0	0	3	0
Emticicia	10 5	10 7	77	14	85	40	17	41	56
Ensifer	40	61	92	0	140	187	92	53	73
Enterobacter	8	5	0	0	0	0	0	0	0
Exiguobacterium	12	17	18	0	0	0	38	4	6
Falsirhodobacter	1	0	5	0	8	0	8	0	5
Ferrovibrio	24	27	11	0	41	25	7	43	15
Ferruginibacter	37	88	57	0	58	24	9	72	34
FFCH7168	3	0	2	4	6	7	0	4	6
Fictibacillus	2	0	0	0	9	8	0	0	0
Fimbriimonas	5	11	14	4	8	9	14	36	27
Flavisolibacter	0	18	6	6	17	9	3	13	13
Flavitalea	0	0	5	6	10	22	9	9	11
Flavobacterium	53 8	73 4	56 1	245	374	171	706	204 3	975
Fluviicola	4	8	2	0	12	2	0	12	21
Gaiella	20	58	54	18	94	51	35	111	119
Geminicoccus	0	0	6	3	0	0	0	3	4
Gemmatimonas	15	6	16	0	5	0	2	20	6
Geodermatophilus	38	25	12	0	60	18	16	9	16
Georgenia	8	6	0	0	4	0	7	10	0
Glutamicibacter	50	55	25	19	26	30	288	0	103
Gracilibacillus	0	2	6	0	6	4	6	4	0
Haliangium	5	3	0	0	14	9	0	0	0
Haloactinopolyspora	7	0	0	0	10	0	0	0	0
Hassallia	0	7	4	7	43	29	0	4	0
Herminiimonas	6	4	0	0	0	0	0	22	25
Herpetosiphon	0	1	8	2	8	36	0	19	8
Hirschia	31	30	27	10	83	89	6	8	6
Hydrogenophaga	74	70	73	66	258	151	18	32	25
Hyphomicrobium	28	30	32	0	72	52	0	33	27
Iamia	68	63	76	84	101	73	43	70	107
Ilumatobacter	26	6	26	29	66	85	25	45	30
IMCC26207	5	0	0	0	7	10	0	0	0
Irregularibacter	0	3	2	0	8	0	2	0	0
IS_44	3	0	7	0	26	21	0	0	0
Isoptericola	17	0	0	18	0	0	0	0	0
Jatrophihabitans	0	0	0	0	0	3	0	10	5
Kaistia	0	1	5	0	12	9	0	3	5
Kocuria	17	13	27	0	24	17	39	30	38
Kribbella	5	5	6	8	7	12	16	11	12
Labrys	0	2	0	4	1	1	6	0	0
Lacibacter	4	3	0	16	12	6	16	0	0
Lacunisphaera	5	10	7	20	13	15	2	8	2
Lautropia	0	5	0	0	18	13	3	10	9
Lechevalieria	53	35	46	25	50	33	125	31	26
Legionella	3	0	5	0	3	6	2	0	0
Limibaculum	31	17	47	0	21	33	15	50	33
Limnobacter	33	15	24	0	0	0	4	2	6
Litorilinea	0	1	0	0	0	5	0	7	0

Longispora	5	4	7	0	23	18	0	1	1
Luteimonas	0	2	0	0	0	3	0	50	21
Luteitalea	0	2	9	0	14	0	0	0	0
Lysinibacillus	22	23	36	0	30	32	16	45	32
Lysobacter	245	167	271	144	469	301	116	104	105
Mariniflexile	4	3	9	30	6	4	0	21	10
Marmoricola	51	43	45	45	75	93	87	28	51
Massilia	15	36	4	0	0	15	26	3	0
Melghirimyces	0	0	0	0	0	0	0	4	9
Mesorhizobium	91	94	129	57	182	136	123	201	180
Methanobrevibacter	0	0	2	0	4	12	1	7	0
Methanoculleus	5	0	0	0	5	20	0	4	0
Methylobacillus	34	17	27	108	74	38	29	36	14
Methylobacter	2	4	1	0	6	0	0	0	0
Methyloceanibacter	0	3	0	0	17	10	0	4	0
Methylophilus	10	8	16	5	18	12	7	13	28
Methylotenera	92	85	96	38	220	121	106	23	66
Microbacterium	551	406	675	172	577	633	816	531	597
Microlunatus	0	0	0	5	22	22	20	0	17
Micromonospora	9	27	7	0	22	15	0	21	34
Microvirga	58	67	56	8	104	69	78	85	130
MM2	7	9	5	12	8	0	33	9	11
MND1	11	10	12	0	29	20	5	0	0
Modestobacter	7	8	41	0	7	7	8	12	43
Mucilaginibacter	0	0	0	0	1	0	0	13	9
Mycobacterium	107	92	146	47	146	142	198	175	219
Nannocystis	0	7	14	0	35	41	7	17	11
Niastella	0	4	14	0	24	8	3	15	20
Nitrosospira	5	4	0	0	0	8	0	5	0
Nitrospira	10	9	9	4	29	20	9	3	16
Nocardia	4	3	6	8	12	12	5	7	22
Nocardioides	695	479	784	644	1233	1360	1837	874	1276
Nonomuraea	11	10	0	0	9	10	13	0	29
Nordella	8	17	19	8	15	27	13	16	15
Not_Assigned	2676	2728	3436	1020	5693	4424	2716	4416	4342
Novibacillus	4	4	12	0	13	4	0	0	3
Noviherbaspirillum	0	0	3	0	5	3	0	0	0
Novosphingobium	0	21	13	27	34	22	23	28	29
Oceanicella	0	0	0	0	0	5	0	8	0
Oceanobacillus	9	7	15	0	0	10	0	0	0
Ochrobactrum	0	0	9	0	5	0	10	0	7
Ohtaekwangia	16	13	13	0	49	16	6	33	14
Ornithinibacillus	0	0	11	0	13	7	0	0	0
Ornithinicoccus	15	6	23	0	10	7	1	25	14
Ornithinimicrobium	0	0	18	0	6	0	5	26	3
Others	18	21	19	10	42	37	21	44	21
Oxalophagus	5	0	0	2	0	0	0	12	0
Paenarthrobacter	103	77	191	44	80	102	131	80	135

Paenibacillus	9	20	59	0	26	22	24	52	61
Paeniclostridium	0	5	7	0	17	9	4	8	0
Paenisporosarcina	5	0	0	0	12	0	0	5	0
Pajaroellobacter	1	0	0	0	0	0	0	0	9
Paracoccus	7	12	20	0	16	26	42	32	25
Parasegetibacter	0	2	0	4	5	0	0	2	1
Patulibacter	3	3	1	0	0	4	0	2	6
Paucisalibacillus	0	0	10	0	18	0	0	11	0
Pedobacter	407	457	432	93	350	332	1339	406	647
Pedococcus_Phycicoccus	17	0	10	0	0	0	10	0	14
Pedomicrobium	9	21	19	0	72	21	6	26	15
Phaselicystis	7	6	4	0	0	6	3	12	4
Phenylobacterium	61	28	52	41	77	44	16	64	29
Phyllobacterium	0	2	0	8	9	10	27	4	0
Pilimelia	0	0	0	0	14	7	0	0	0
Planococcus	48	37	112	0	21	24	114	69	86
Polaromonas	14	13	20	20	55	23	20	12	27
Pontibacter	0	0	0	8	5	0	6	8	4
Porphyrobacter	0	0	0	0	0	0	0	26	18
Promicromonospora	16	3	7	20	0	15	34	11	0
Pseudoflavitalea	0	0	4	9	24	2	0	0	0
Pseudolabrys	13	0	10	8	28	12	10	36	21
Pseudomonas	69	58	88	18	68	76	30	17	27
Pseudonocardia	25	15	23	0	21	19	10	35	59
Pseudorhodoferax	10	6	15	7	6	10	0	0	15
Pseudorhodoplanes	0	0	0	0	12	11	0	0	0
Pseudoxanthomonas	61	59	62	63	150	117	263	107	138
Psychrobacillus	2	5	7	0	0	0	0	13	9
Quadrisphaera	9	0	0	0	15	5	8	27	24
Ramlibacter	0	0	0	0	11	18	0	0	7
Rathayibacter	0	0	0	0	0	0	10	0	12
Reyranella	6	15	18	0	40	50	14	30	30
Rhabdanaerobium	0	0	1	0	5	0	2	0	5
Rheinheimera	5	12	18	13	5	0	4	0	20
Rhizobacter	0	8	0	0	0	0	6	9	0
Rhizobiumsphaerophysaegroup	54	101	82	0	54	53	62	174	96
Rhizocola	3	3	2	0	2	0	0	15	9
Rhodanobacter	0	0	0	0	0	0	3	88	79
Rhodobacter	0	4	8	0	0	5	0	0	0
Rhodococcus	24	27	34	9	23	64	41	33	100
Rhodocytophaga	0	0	0	16	22	13	9	0	4
Rhodomicrobium	0	3	13	0	12	7	3	13	5
Rhodoplanes	0	0	0	0	12	9	0	0	0
Romboutsia	7	13	0	0	45	22	5	18	22
Roseomonas	5	5	0	6	5	3	23	2	12
Rubellimicrobium	0	3	4	7	19	11	14	19	16
Rubrobacter	0	6	0	0	0	31	19	6	0
Rurimicrobium	0	0	2	0	7	4	0	5	0
Saccharomonospora	9	4	14	0	5	9	4	6	3
Sandaracinus	5	2	2	4	6	6	1	1	8
Sedimentibacter	0	0	0	0	7	3	0	0	0
Serratia	0	0	0	10	0	2	0	0	0

Shinella	9	12	35	0	12	19	13	36	20
Skermanella	39	41	37	8	65	69	77	80	72
Solibacillus	0	9	0	0	0	0	0	13	9
Solirubrobacter	18	38	0	18	0	72	0	89	37
Sphingobacterium	3	10	5	4	9	13	16	21	39
Sphingobium	261	320	281	107	395	231	490	272	408
Sphingomonas	271	261	273	155	484	432	356	468	687
Sphingopyxis	185	192	226	75	390	323	479	239	364
Sporacetigenium	0	0	4	0	0	4	0	0	4
Sporichthya	0	0	0	0	2	0	47	0	33
Staphylococcus	6	0	0	5	23	62	6	0	4
Stenotrophobacter	9	14	11	0	35	30	0	21	0
Stenotrophomonas	55	46	112	0	24	36	28	7	14
Steroidobacter	31	74	48	93	19	61	12	63	54
Streptomyces	221	196	244	342	239	310	355	412	390
Subgrou10	10	4	3	15	32	8	0	0	0
Subsaxibacter	5	6	8	0	7	9	0	7	9
SWB02	11	7	6	6	4	5	2	5	0
Tahibacter	80	62	102	49	197	112	48	133	95
Taibaiella	11	25	13	2	50	19	32	82	66
Tardiphaga	0	0	0	0	14	21	0	0	0
Terrabacter	0	0	0	10	12	10	16	0	12
Terribacillus	14	11	10	0	5	8	10	11	8
Terrimonas	17	16	25	14	138	112	13	0	0
Thalassobacillus	0	0	0	0	10	6	0	0	6
Thermomonas	24	17	28	0	62	20	58	5	41
TM7a	50	51	33	22	89	37	264	159	316
Tsukamurella	0	0	0	0	0	0	18	0	7
Tumebacillus	0	0	24	0	5	7	0	7	15
Turicibacter	11	14	29	0	57	34	18	26	41
unidentified67_14	4	0	0	0	4	5	0	0	0
unidentifiedAcidobacteriales	3	0	0	0	0	7	0	0	0
unidentifiedActinomarinales	2	9	8	0	18	15	6	3	6
unidentifiedAlphaproteobacteria	0	5	3	0	0	0	0	0	9
unidentifiedChitinophagaceae	2	5	8	0	22	8	0	30	20
unidentifiedChloroplast	80	99	78	63	39	45	83	64	62
unidentifiedD05_2	0	0	4	0	3	0	0	4	7
unidentifiedGaiellales	0	0	0	4	5	3	4	0	0
unidentifiedGemmatimonadaceae	21	10	21	14	19	12	0	26	34
unidentifiedMicrotrichales	7	0	5	0	13	11	7	20	16
unidentifiedMitochondria	4	10	16	82	0	28	15	9	7
unidentifiedRhodanobacteraceae	24	18	31	0	11	6	0	12	0
unidentifiedRhodocyclaceae	116	120	116	67	176	126	83	72	86
unidentifiedS085	0	0	0	0	0	3	0	8	0
unidentifiedSandaracinaceae	112	154	77	73	82	52	0	126	33
unidentifiedSC_I_84	12	24	29	0	27	18	6	31	43
unidentifiedSericytochromatia	0	0	0	0	0	0	2	0	11

unidentifiedSolirubrobacteraceae	0	0	2	0	0	0	0	3	9
unidentifiedSteroidobacteraceae	0	6	0	0	0	2	2	0	8
unidentifiedVicinamibacterales	0	10	13	0	0	0	0	29	9
unidentifiedXanthobacteraceae	7	11	13	0	34	24	13	17	6
UTBCD1	0	3	0	2	8	0	0	4	0
Variovorax	39	29	23	6	58	60	110	22	67
Virgisporangium	6	0	7	0	9	0	4	6	0
Vitellibacter	6	13	10	0	0	0	5	18	7
Williamsia	0	0	0	0	0	0	4	0	7
Woeseia	0	1	0	0	7	0	5	4	0
YC_ZSS_LKJ147	4	6	2	6	0	2	0	5	5

Table S4. Analysis of variance (ANOVA) for the relative abundaces of the top 10 phyla and genera.

*** p<0.001; * p<0.05.

		**F-statistic**	**p-value**	**sig. level**
Top Phyla	Acidobacteriota	0.5372	0.6101	-
	Actinobacteriota	0.7671	0.505	-
	Bacteroidota	5.3769	0.04593	*
	Chloroflexi	0.6973	0.5342	-
	Firmicutes	2.121	0.201	-
	Gemmatimonadota	0.3015	0.7503	-
	Myxococcota	1.6544	0.2678	-
	Patescibacteria	9.771	0.01296	*
	Proteobacteria	33.1353	0.0005722	***
Top Genera	Bacillus	2.6039	0.1534	-
	Cellvibrio	7.4292	0.0238	*
	Flavobacterium	2.2761	0.1838	-
	Microbacterium	1.2184	0.3597	-
	Nocardioides	2.2073	0.1912	-
	Pedobacter	1.0694	0.4007	-
	Sphingobium	0.1525	0.8618	-
	Sphingomonas	0.8803	0.4621	-
	Streptomyces	0.9356	0.4429	-

Table S5. T-test of significance on the predicted functions according to the COG library for prokaryotic communities in the rhizosphere of *Petromarula pinnata* at three different altitudes in eastern Crete (**p<0.01; * p<0.05).

**Function**	**Description**	**Relative abundances**				**p-value**	**Significance**	**Category**	**Code**
		**Intermediate**		**Higher**					
		mean	stdev	mean	stdev				
COG1028	NAD(P)-dependent dehydrogenase, short-chain alcohol dehydrogenase family	0.00421	0.00006	0.00409	0.00004	0.049	*	Lipid transport and metabolism	I
COG0673	Predicted dehydrogenase	0.00223	0.00003	0.00239	0.00004	0.005	**	General function	R
COG0625	Glutathione S-transferase	0.00193	0.00008	0.00159	0.00002	0.015	*	Posttranslational modification, protein turnover, chaperones	O
COG0683	ABC-type branched-chain amino acid transport system, periplasmic component	0.00184	0.00006	0.00163	0.00001	0.025	*	Amino acid transport and metabolism	E
COG1733	DNA-binding transcriptional regulator, HxlR family	0.00131	0.00011	0.00153	0.00007	0.046	*	Transcription	K
COG0715	ABC-type nitrate/sulfonate/bicarbonate transport system, periplasmic component	0.00153	0.00003	0.00146	0.00001	0.041	*	Transcription	K
COG0366	Glycosidase	0.00109	0.00002	0.00117	0.00002	0.012	*	Carbohydrate transport and metabolism	G
COG1473	Metal-dependent amidase/aminoacylase/carboxypeptidase	0.00104	0.00003	0.00111	0.00002	0.033	*	General function	R
COG0776	Bacterial nucleoid DNA-binding protein	0.00104	0.00003	0.00095	0.00003	0.028	*	Replication, recombination and repair	L
COG0402	Cytosine/adenosine deaminase or related metal-dependent hydrolase	0.00100	0.00001	0.00092	0.00002	0.003	**	Nucleotide transport and metabolism	F

		**Lower**	**Intermediate**						
		mean	stdev	mean	stdev				
COG0204	1-acyl-sn-glycerol-3-phosphate acyltransferase	0.00117	0.00002	0.00122	0.00001	0.020	*	Lipid transport and metabolism	I
COG0842	ABC-type multidrug transport system, permease component	0.00104	0.00002	0.00108	0.00002	0.039	*	Defense mechanisms	V
COG0402	Cytosine/adenosine deaminase or related metal-dependent hydrolase	0.00097	0.00001	0.00100	0.00001	0.025	*	Nucleotide transport and metabolism	F
		**Lower**	**Higher**						
		mean	stdev	mean	stdev				
COG0642	Signal transduction histidine kinase	0.00456	0.00011	0.00424	0.00004	0.026	*	Signal transduction mechanisms	T
COG0596	Pimeloyl-ACP methyl ester carboxylesterase	0.00457	0.00011	0.00432	0.00005	0.049	*	Coenzyme transport and metabolism	H
COG0745	DNA-binding response regulator, OmpR family, contains REC and winged-helix (wHTH) domain	0.00455	0.00010	0.00421	0.00003	0.023	*	Transcription	K
COG0451	Nucleoside-diphosphate-sugar epimerase	0.00427	0.00016	0.00389	0.00006	0.044	*	Cell wall/membrane/envelope biogenesis	M
COG0515	Serine/threonine protein kinase	0.00337	0.00027	0.00261	0.00011	0.025	*	Signal transduction mechanisms	T
COG1028	NAD(P)-dependent dehydrogenase, short-chain alcohol dehydrogenase family	0.00417	0.00003	0.00409	0.00004	0.050	*	Lipid transport and metabolism	I
COG1357	Uncharacterized protein YjbI, contains pentapeptide repeats	0.00283	0.00032	0.00185	0.00013	0.021	*	Function unknown	S
COG4636	Endonuclease, Uma2 family (restriction endonuclease fold)	0.00271	0.00033	0.00171	0.00014	0.021	*	General function	R
COG2814	Predicted arabinose	0.00327	0.00007	0.00350	0.00006	0.012	*	Carbohydrate transport	G

	efflux permease, MFS family							and metabolism	
COG1595	DNA-directed RNA polymerase specialized sigma subunit, sigma24 family	0.00290	0.00008	0.00329	0.00009	0.006	**	Transcription	K
COG1309	DNA-binding transcriptional regulator, AcrR family	0.00296	0.00009	0.00325	0.00005	0.017	*	Transcription	K
COG1012	Acyl-CoA reductase or other NAD-dependent aldehyde dehydrogenase	0.00251	0.00011	0.00286	0.00004	0.020	*	Energy production and conversion	C
COG0664	cAMP-binding domain of CRP or a regulatory subunit of cAMP-dependent protein kinases	0.00253	0.00012	0.00227	0.00005	0.046	*	Signal transduction mechanisms	T
COG1131	ABC-type multidrug transport system, ATPase component	0.00249	0.00006	0.00269	0.00008	0.026	*	Defense mechanisms	V
COG0589	Nucleotide-binding universal stress protein, UspA family	0.00237	0.00011	0.00205	0.00007	0.019	*	Signal transduction mechanisms	T
COG1960	Acyl-CoA dehydrogenase related to the alkylation response protein AidB	0.00216	0.00013	0.00260	0.00007	0.015	*	Lipid transport and metabolism	I
COG1846	DNA-binding transcriptional regulator, MarR family	0.00211	0.00014	0.00258	0.00005	0.020	*	Transcription	K
